# Influence of Herbicide Triasulfuron on Soil Microbial Community in an Unamended Soil and a Soil Amended with Organic Residues

**DOI:** 10.3389/fmicb.2017.00378

**Published:** 2017-03-08

**Authors:** Eva Pose-Juan, José M. Igual, María J. Sánchez-Martín, M. S. Rodríguez-Cruz

**Affiliations:** Environmental Degradation and its Remediation, Instituto de Recursos Naturales y Agrobiología de Salamanca, Spanish National Research Council (IRNASA-CSIC)Salamanca, Spain

**Keywords:** herbicide, soil, amendments, dissipation, microbial function, microbial structure

## Abstract

The effect of organic amendments and pesticides on a soil microbial community has garnered considerable interest due to the involvement of microorganisms in numerous soil conservation and maintenance reactions. The aim of this work was to assess the influence on a soil microbial community of the simultaneous application of the herbicide triasulfuron at three doses (2, 10, and 50 mg kg^-1^), with an organic amendment [sewage sludge (SS) or green compost (GC)]. Dissipation kinetics, soil microbial biomass, dehydrogenase activity (DHA) and respiration, and the profile of phospholipid fatty acids (PLFAs) extracted from the soil, were determined in unamended (S) soil and amended (S+SS and S+GC) ones. Triasulfuron dissipation followed the single first-order kinetics model. Half-life (DT_50_) values were higher in the amended soils than in the unamended one for the 10 and 50 mg kg^-1^ doses. The dissipation rates were lower in the S+GC soil for the three herbicide doses applied. In general, soil biomass, DHA and respiration values increased in SS- and GC-amended soils compared to the unamended one. DHA values decreased (S and S+SS) or increased (S+GC) with the incubation time of soil with herbicide at the different doses applied. Respiration values increased with the herbicide doses applied and decreased with the incubation time, although maximum values were obtained for soils treated with the highest dose after 70 days of incubation. PLFA analysis indicated different effects of triasulfuron on the soil microbial community structure depending on the organic amendments. While the increasing triasulfuron doses resulted in deeper alterations in the S soil, the time after triasulfuron application was the most important variation in the S+SS and S+GC soils. The overall results indicate that the soil amendment has an effect on herbicide dissipation rate and the soil microbial community. Initially, a high dose of triasulfuron had detrimental effects on the soil microbial community, which is important in the case of the long-term use of this compound.

## Introduction

Soil biodiversity is threatened by some agricultural practices and soil contamination, among other phenomena, with consequences for the soil ecosystem and its functions and, ultimately, for soil quality. Changes in soil biodiversity could affect crop yields, nutrient cycling, soil fertility, bioremediation capacity, soil formation and structure, etc., which should be avoided to ensure soil protection (European Commission [EC], 2006). There is limited information about the effect of agricultural practices involving the application of organic amendments and pesticides on microbial communities and/or soil biodiversity (Scelza et al., 2008; Rodríguez-Liébana et al., 2014; Pose-Juan et al., 2015a,b; Singh et al., 2016). Further investigation is required to evaluate the impact these agricultural management practices have on soil conservation and maintenance in order to prevent its degradation.

The impact of pesticides on a soil microbial community depends on their toxicity, which determines hazard, and on their fate in the soil, which is governed by several processes such as adsorption, leaching, run-off, degradation, volatilization, plant uptake, etc. These processes control the amount of pesticide in the soil that is bioavailable for affecting microorganisms (Jacobsen and Hjelmsø, 2014), and they could be modified by the application of organic residues (Herrero-Hernández et al., 2011).

On the other hand, the application of organic amendments to soil is a current strategy for increasing nutrient and organic matter (OM) content to improve its fertility, avoid its degradation, and contribute to its quality (Bastida et al., 2015). Sewage sludge (SS) from urban wastewater treatment plants and green compost (GC) from plant pruning in parks and gardens are some of the organic residues used in agriculture. In Spain, 914929 t of SS (81% of the total generated) was applied in agriculture in 2012, and the amount of GC recorded in Spain was 256000 t in 2013 (Ministerio de Agricultura Alimentación y Medio Ambiente [MAGRAMA], 2014).

The dissipation of pesticides in soils amended with different organic residues under laboratory or field conditions has been reported (Marín-Benito et al., 2014; Herrero-Hernández et al., 2015; Gámiz et al., 2016). Other studies have addressed the changes in soil microbial structure and diversity after pesticide and organic residue application (Singh et al., 2014; Feld et al., 2015; Pose-Juan et al., 2015a; Álvarez-Martín et al., 2016). However, information is limited on the abundance and performance of soil microbial communities following the simultaneous application of pesticides and organic residues, due to the large number of pesticides and organic residues used in agriculture. In general, the impact on the soil of an agronomic dose of pesticides is included in these studies, with the application of increasing doses of pesticides being scarcely considered (Karpouzas et al., 2014; Xu et al., 2014).

Concentrations of pesticides that are higher than the field rate could be found in soils due to repeated pesticide treatment in monocultural practices or to accidental spillages (Perucci et al., 1999). Accordingly, it is of great interest to study the effects of increasing pesticide doses on soil microbial communities, using different technical approaches to assess the potential side-effects on non-target microorganisms.

Triasulfuron (1-[2-(2-chloroethoxy) phenylsulfonyl]-3-(4-methoxy-6-methyl-1,3,5-triazin-2-yl) urea) is a sulfonylurea herbicide applied at rates of 15–25 g ha^-1^. It controls a wide spectrum of weeds in crops such as wheat, oats and barley in pre- or post-emergence. It is an herbicide with low toxicity for humans and low-to-moderate ecotoxicity (Pesticide Properties Database [PPDB], 2015). The major pathways of triasulfuron degradation are microbial degradation and chemical hydrolysis, being influenced by soil pH, OM, clay content, organic and inorganic fertilizers, and climatic conditions (rainfall and temperature) (Walker and Welch, 1989; Stork, 1995; Sarmah et al., 1998; Menne and Berger, 2001; Hollaway et al., 2006). In laboratory studies (aerobic conditions in the dark), triasulfuron has recorded moderate-to medium-persistence (European Food Safety Authority [EFSA], 2015). There is limited information on its dissipation and effect on a soil microbial community in unamended or amended soils (Said-Pullicino et al., 2004; Sofo et al., 2012).

The aim of this work was to assess the influence of the simultaneous application of the herbicide triasulfuron with an organic amendment (SS or GC) on a soil microbial community. The dissipation kinetics of triasulfuron applied at three doses, 2, 10, and 50 mg kg^-1^ were studied in unamended S and S+SS and S+GC soils. Soil microbial biomass, dehydrogenase activity (DHA) and respiration, and the profile of phospholipid fatty acids (PLFAs) extracted from the soil, were determined at different incubation times of the soil-organic amendment-herbicide.

## Materials and Methods

### Herbicide and Chemicals

Triasulfuron pure standard from PESTANAL^®^ was supplied by Sigma–Aldrich Química SA (Madrid, Spain) (>99% purity). It is a weak acid (pKa = 4.64) with a water solubility of 815 mg L^-1^ (20°C), and a log *K*_ow_ of -0.59 (pH 7, 20°C) (Pesticide Properties Database [PPDB], 2015).

HPLC grade acetonitrile and chloroform anhydrous (>99% purity) were supplied by VWR International Eurolab (Barcelona, Spain). Ammonium sulfate, potassium sulfate (≥99% purity), ninhydrin reagent solution, 2,3,5-triphenyltetrazolium chloride (TTC) and 2,3,5-triphenylformazan (TPF) were supplied by Sigma–Aldrich Química SL (Madrid, Spain).

### Organic Residues and Soil

Green compost (GC) consisting of wastes from pruning was supplied by the municipal authority in Salamanca (Spain). SS from an urban waste treatment plant and stabilized by anaerobic digestion was supplied by Aqualia SA (Salamanca, Spain). Their characteristics were determined in samples that had previously been air dried, homogenized and sieved (<2 mm) (**Table 1**). The pH was determined in a residue/water suspension (1/2.5 w/v ratio). Organic carbon (OC) content was determined by oxidation (Walkley-Black method). Dissolved organic carbon (DOC) was determined in a suspension of residue (1/100 w/v ratio) in Milli-Q ultrapure water after residue shaking (24 h at 20°C), centrifugation (20 min at 10000 rpm), and filtering (Minisart NY 25 filter 0.45 μm, Sartorius Stedim Biotech, Germany) using a Shimadzu 5050 OC analyzer (Shimadzu, Columbia, MD, USA). Total N was determined by the Kjeldahl method. The cation exchange capacity (CEC) was determined by the ammonium acetate method, and the cations were determined by atomic absorption spectrophotometry (Sparks, 1996).

**Table 1:** Characteristics of green compost (GC) and sewage sludge (SS) and unamended (S) and amended (S+GC and S+SS) soils given on a dry weight basis.

	GC	SS	S	S+GC	S+SS
pH	7.1	6.7	6.3	7.0	6.2
OC (%)	8.06	27.0	0.49	1.50	2.20
DOC^a^ (mg g^-1^)	1.02	21.7	0.05	0.07	0.68
N (%)	0.79	4.76	0.04	0.18	0.35
C/N	10.2	5.67	12.2	8.33	6.28
CEC^b^ (Cmol kg^-1^)	18.7	64.5	4.38	6.87	7.95
Na (Cmol kg^-1^)	0.27	1.22	0.03	0.03	0.02
K (Cmol kg^-1^)	56.4	4.99	0.66	2.78	0.78
Ca (Cmol kg^-1^)	28.9	24.5	6.96	17.2	10.8
Mg (Cmol kg^-1^)	3.83	14.2	2.40	3.16	4.33
Clay (%)	-	-	10.7	-	-
Silt (%)	-	-	5.9	-	-
Sand (%)	-	-	83.4	-	-

*^a^Dissolved organic carbon, ^b^Cationic exchange capacity.*

The soil was taken from the surface horizon (0–30 cm) on an agricultural farm (Toro, NW-Spain). It is a Typic Xerorthent (Soil Survey Staff, 2006) with a sandy loam texture (84.9, 5.78, and 9.29% of sand, silt, and clay, respectively). There had been no application of triasulfuron to the field over at least the previous 10 years. The soil was sieved (<2 mm) and stored at 4°C until further use. Soil characteristics (pH, OC, DOC, and CEC) were determined as described above (**Table 1**). Inorganic carbon content was determined as CaCO_3_ with a Bernard calcimeter (Sparks, 1996).

The amended soils were prepared in October 2012 by uniformly mixing soil with GC or SS *in situ* in the field at a rate of 50 t ha^-1^ (considering a soil depth of ∼5 cm and a soil density of 1.3 g cm^-3^). The unamended soil is termed S hereafter, while the soil amended with GC or SS is termed S+GC and S+SS, respectively. Undisturbed soil samples of all the treatments (∼30 kg) were collected from the field (0–30 cm) and incubated outside under environmental conditions in 60 cm × 40 cm × 25 cm trays at IRNASA (Salamanca, Spain) over the experimental period. Soil samples were sieved (<2 mm) prior to their use in dissipation experiments. Their characteristics are included in **Table 1**.

### Dissipation Microcosms

A solution of 5000 mg L^-1^ of triasulfuron in methanol was used as a first standard to prepare solutions of the herbicide in Milli-Q ultrapure water. A 10 mL volume of adequate concentration was then added to 500 g of fresh weight of S, S+GC, and S+SS to obtain a triasulfuron concentration of 2, 10, or 50 mg kg^-1^ dry soil. Samples of soil+triasulfuron were incubated at 20°C in the dark. The moisture content of the soil samples was previously adjusted to 40% of the maximum soil water-holding capacity, and it was maintained by adding sterile Milli-Q ultrapure water when necessary. Each soil treatment was prepared in duplicate. A sample of the sterilized S was also prepared by autoclaving soil at 120°C for 1 h on three consecutive days. The sterilized S was treated with the herbicide, and incubated as indicated above, and these samples were used as controls to check the chemical degradation of triasulfuron. Finally, the soils for microbiological control were prepared by adding only sterile Milli-Q ultrapure water. All the soils were thoroughly stirred with a sterilized spatula, and all the steps were performed in a sterile cabinet. Soil samples were taken at day 0 for pesticide analysis, and thereafter repeatedly at different time intervals (0, 7, 15, 22, 28, 35, 42, 49, 56, 70, 85, 99, 114, 132, 141, 147, 155, and 170 days), depending on the dissipation rate of triasulfuron in each soil treatment.

### Extraction and Determination of Triasulfuron

Duplicate 5 g samples of each duplicate treatment were taken at each sampling time and shaken at 20°C for 2 h with 10 mL of methanol:acetone (1:1) in glass tubes. The samples were then sonicated for 1 h and centrifuged at 5045 *g* for 15 min, and the herbicide extracts were filtered in a Minisart NY 25 filter (Sartorius Stedim Biotech, Germany) to remove particles >0.45 μm. For the determination of the herbicide and its possible metabolites, a volume of the extract was transferred to a glass vial for analysis. The recoveries of the extraction method were determined by spiking three unamended and amended soil samples with analytical grade pesticide to a final concentration of 2 mg kg^-1^, performing the extraction procedure as described above. The mean recovery values were 92% for S, 80% for S+GC and 70% for S+SS.

Triasulfuron was quantified by HPLC with diode array (DAD) and mass spectrometer (MS) detectors (Waters Associates, Milford, MA, USA), using Empower software as the data acquisition and processing system. The MS parameters were as follows: capillary voltage, 3.1 kV; source temperature, 120°C; the desolvation temperature and the desolvation gas flow were set at 300°C and 400 L h^-1^, respectively, and the cone gas flow at 60 L h^-1^. The analytical column was a Luna PFP (2) (150 × 4.6 mm i.d., 3.0 μm) (Phenomenex, Torrance, CA, USA). The mobile phase was 90:10 (v/v) acetonitrile/water (1% formic acid). The flow rate of the mobile phase was 0.4 mL min^-1^, and the sample injection volume was 10 μL. The retention time was 5.1 min. Quantitative analysis was performed using the peak area of the compound obtained from the total ion chromatogram (TIC) in SIM mode. The molecular ion (m/z) corresponding to triasulfuron in the positive ionization mode [M + H]^+^ was 402.8. Calibration was performed from 0.5 to 25 μg mL^-1^ with a correlation coefficient of ≥0.99, and the limit of detection (LOD) and limit of quantification (LOQ) were >0.012 and >0.113 μg mL^-1^, respectively.

### Soil Biochemical Parameters and Microbial Structure Analysis

At the beginning of the dissipation experiments (0 days), and at 70 and 150 days, soil biomass-N, DHA, respiration and PLFAs were measured in unamended and amended soils (control (untreated) and herbicide treated soils).

Microbial biomass-N was extracted using the chloroform fumigation-extraction technique (Vance et al., 1987). Ninhydrin-reactive N released by fumigation was converted to biomass-C using a conversion factor of 20.6 (Joergensen and Brookes, 1990). The results are expressed in dry soil and are the mean of two replicates. Soil DHA was determined following the Tabatabai method (Tabatabai, 1994). Soil respiration was determined by measuring O_2_ absorption by microorganisms in 50 g portions of soil incubated at 25°C for 96 h, using an OxiTop Control BM6 containers with an OxiTop Control OC 110 measurement system (WTW, Weilheim, Germany).

The microbial community composition of the unamended and amended soil samples was determined using PLFA analysis, as described in Pose-Juan et al. (2015a). Briefly, samples were freeze-dried, and 3 g of dry material was used for lipid extraction. Lipids were extracted with a one-phase chloroform-methanol-phosphate buffer solvent. Phospholipids were separated from non-polar lipids and converted to fatty acid methyl esters before analysis. Quantification was performed using an Agilent 7890 gas chromatograph (Agilent Technologies, Wilmington, DE, USA) equipped with a 25-m Ultra 2 (5% phenyl)-methylpolysiloxane column (J&W Scientific, Folsom, CA, USA) and with a flame ionization detector (FID). PLFAs were identified using bacterial fatty acid standards and software from the Microbial Identification System (Microbial ID, Inc., Newark, DE, USA).

### Data Analysis

The dissipation kinetics for triasulfuron was fitted to a single first-order (SFO) kinetic model or first-order multicompartment (FOMC) model, known also as the Gustafson and Holden model. For the selection of the kinetic model that best describes the dissipation results, FOCUS work group guidance recommendations were followed (FOCUS, 2006). The coefficient of determination (*r*^2^) and the chi-square (χ^2^) test were calculated as indicators of the goodness of fit. Values for the time to 50% dissipation (DT_50_) were used to characterize the decay curves and compare variations in dissipation rates. The parameters of the kinetic models were estimated using the Excel Solver add-in package (FOCUS, 2006).

Standard deviation (SD) was used to indicate variability among replicates. Data were analyzed by two-way ANOVA being the main factors soil treatments, incubation times and triasulfuron doses. When significant interactions were observed between these factors the Fisher’s least significant differences (LSD), at a confidence level of 95%, were used to determine significant differences between means and evaluate the effects of the different treatments on DT_50_ values and the soil microbial biomass, DHA, as well as respiration. SPSS Statistics v22.0 software for Windows (IBM Inc., Chicago, ILL, USA) was used.

Phospholipid fatty acid data (%mol) were processed using multivariate principal component analysis (PCA) and redundancy analysis (RDA) and a one-way ANOVA with CANOCO v5.04 (Microcomputer Power, Ithaca, NY, USA) and SPSS v21.0 for Windows (IBM Corp., Armonk, NY, USA) programs. Different soil treatments, incubation times and triasulfuron doses were coded as dummy variables, and used as independent variables in the multivariate analyses. Significance in RDA was tested by Monte Carlo permutation tests (999 unrestricted permutations) for the sum of all canonical axes. In univariate comparisons, LSD *post hoc* tests were used to identify those means significantly different at *P* ≤ 0.05. The molar percentage values were arcsin transformed prior to statistical analyses in order to meet normality assumptions.

## Results and Discussion

### Dissipation of Triasulfuron Applied at Different Doses in Soils

**FIGURE 1:**
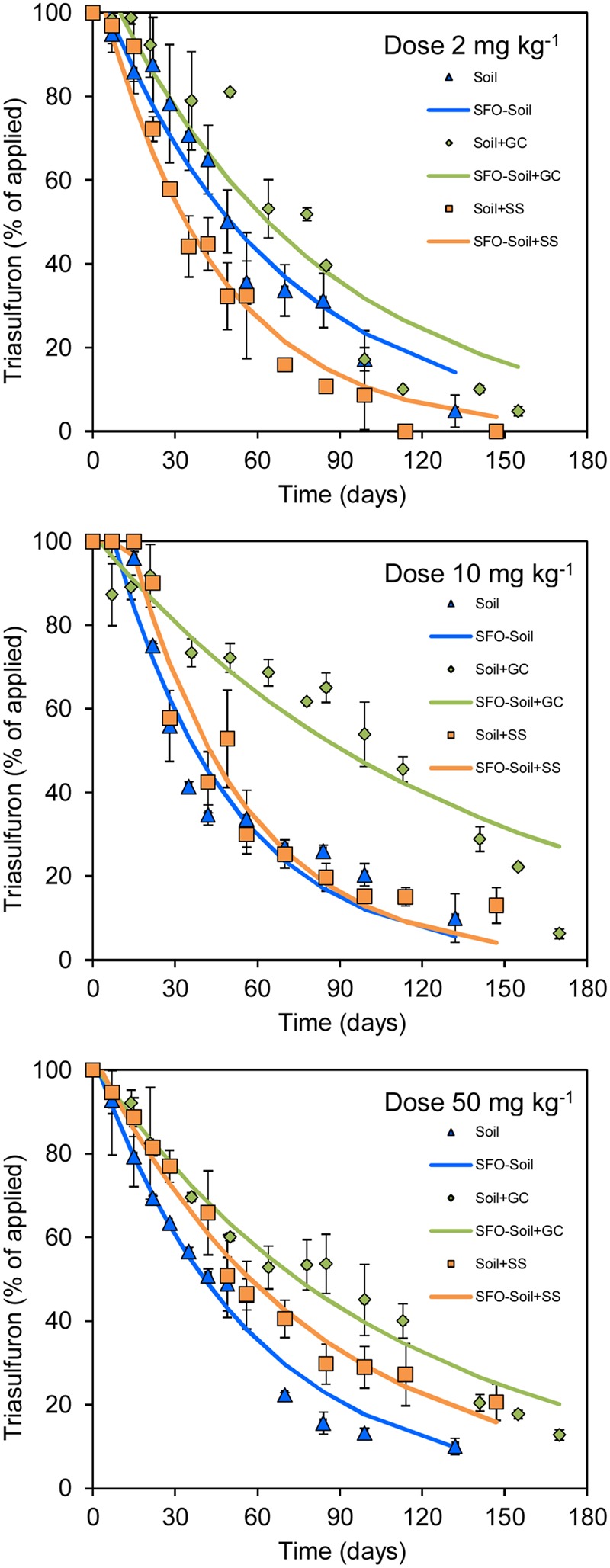
**Dissipation kinetics of triasulfuron applied at doses of 2, 10, and 50 mg kg^-1^ in unamended soil and soil amended with sewage sludge (SS) or green compost (GC).** Bars represent standard deviation (SD) of the mean (*n* = 2).

**Figure 1** shows the dissipation kinetics of triasulfuron applied at doses of 2, 10, and 50 mg kg^-1^ in unamended and amended soils. Data for the remaining percentages of the herbicide as a function of time provided a good fit to the SFO kinetic model (χ^2^ < 15 and *r*^2^ ≥ 0.90), and the kinetic parameters are presented in **Table 2**. In previous studies, the dissipation of triasulfuron in soils has been fitted to a first-order kinetic model (Walker and Welch, 1989; James et al., 1999; Menne and Berger, 2001; Gennari et al., 2008) or to a biphasic one (Said-Pullicino et al., 2004). The dissipation kinetics of triasulfuron applied at 10 mg kg^-1^ in S and S+SS soils recorded an initial lag phase of seven and 15 days, respectively.

**Table 2:** Kinetics parameters for the degradation of triasulfuron in unamended and amended soils obtained from fitting kinetics to a single first order (SFO) model.

Soil sample	Dose (mg kg^-1^ dw)	*k* (days^-1^)	DT_50_ (days)	*r*^2^	χ^2^
Soil	2	0.016	44.6 ± 8.8bc	0.95	9.6
Soil+GC	2	0.012	57.7 ± 0.6d	0.95	13.5
Soil+SS	2	0.024	29.3 ± 2.6a	0.96	11.6
Soil	10	0.023	37.0^a^ ± 2.7ab	0.97	12.8
Soil+GC	10	0.008	88.9 ± 0.2f	0.95	11.0
Soil+SS	10	0.024	44.1^b^ ± 3.4bc	0.97	14.0
Soil	50	0.018	38.8 ± 1.4ab	0.98	6.9
Soil+GC	50	0.010	72.9 ± 6.3e	0.96	6.8
Soil+SS	50	0.013	54.3 ± 8.3cd	0.98	5.3
LSD (95%)			11.03		

*^a^DT_*50*_ value include a lag phase of 7 days; ^b^DT_*50*_ value include a lag phase of 15 days. DT_*50*_ values with the same letter are not significantly different.*

The estimated DT_50_ values of triasulfuron applied at increasing doses (10 and 50 mg kg^-1^) decreased in S and increased in both amended soils (LSD = 11.03, *P* < 0.0001). The DT_50_ values calculated for S (37.0–44.6 days) are consistent with the DT_50_ values reported (7.8–118 days) for the dissipation of triasulfuron in soils (James et al., 1999; European Commission [EC], 2000; Gennari et al., 2008; European Food Safety Authority [EFSA], 2015; Pesticide Properties Database [PPDB], 2015). The DT_50_ values of triasulfuron applied at the lowest dose (2 mg kg^-1^) were different between soils, and followed the order S+SS < S < S+GC. When the herbicide was applied at higher doses (10 and 50 mg kg^-1^), the DT_50_ values followed the order S < S+SS < S+GC (**Table 2**). The higher dissipation rate of triasulfuron at higher doses in S might be attributed to a saturation of adsorption sites and a higher bioavailability of the herbicide to be degraded, as observed for other pesticides (Papadopoulou et al., 2016). However, a decrease in the dissipation rate was also reported when the dose of herbicide increased from 20 to 1000 μg kg^-1^ for triasulfuron (Menne and Berger, 2001) and for other pesticides applied at increasing doses (Vischetti et al., 2000; El-Ghamry et al., 2002; Muñoz-Leoz et al., 2013; Pose-Juan et al., 2015b). The lower pesticide dissipation rate was attributed to the reduced capacity of soil microbial communities to biodegrade them, variations in microbial biomass, and the pesticide’s toxicity for the soil microbial community.

The dissipation rates of triasulfuron in S were higher than in the amended soils for the 10 and 50 mg kg^-1^ doses. The higher DT_50_ values found in amended soils could be explained by their higher adsorption of triasulfuron (Kd = 1.65 mL g^-1^ for S+SS and Kd = 1.18 mL g^-1^ for S+GC) than by S (Kd = 0.94 mL g^-1^) (Abaunza, 2014). A relationship between triasulfuron adsorption and degradation in soil has been reported by Said-Pullicino et al. (2004). These authors studied the degradation of triasulfuron in an unamended soil and in one amended with municipal waste compost, finding an increase in the DT_50_ value of 19 to 23 days, indicating a decrease in the bioavailability and biodegradation of the herbicide adsorbed.

The dissipation rates were higher in S+SS than in S+GC for the three herbicide doses applied. The lower DT_50_ values obtained in S+SS could be explained by the increased adsorption of triasulfuron by this soil with the highest OC content (**Table 1**). Bound residues that are difficult to extract may also be formed, resulting in a higher apparent dissipation, as observed for other herbicides (Marín-Benito et al., 2014). Furthermore, dissipation might be accelerated in SS-amended soils due to a bigger decrease in soil pH than in GC-amended soils. Triasulfuron is a weak acid (pKa = 4.6), and adsorption may increase at a lower pH (Abaunza, 2014) with an increase in the apparent dissipation rate. At a lower pH, the principal modes of triasulfuron degradation are acid hydrolysis and microbial degradation (Sarmah et al., 1998). In this sense, Walker and Welch (1989) found a significant negative correlation between the degradation rates of some sulfonylureas, including triasulfuron, and the pH of unamended soils. In previous studies, triasulfuron dissipation increased when straw was added to the soil (Menne and Berger, 2001), and a relationship was found between soil pH and the accelerated hydrolysis of triasulfuron in straw-amended soils.

In S+GC soil, the DT_50_ value fell when the herbicide application dose increased from 10 to 50 mg kg^-1^, indicating that the herbicide could be more bioavailable for microbial degradation at higher doses due to a decrease in the percentage of herbicide adsorbed at higher concentrations (Abaunza, 2014). The lower dissipation rate of herbicide in S+GC could be due to this residue’s OC content and type of OM. Barriuso et al. (1997) reported that compost inhibited the mineralization of herbicides in the soil through a stronger adsorption of compounds or the incorporation of OM fractions.

The different rates of triasulfuron dissipation in unamended and amended soils indicate that the SS and GC amendments provide microorganisms and OM to the soil that affect the bioavailability of the herbicide in the amended soils.

In sterilized unamended soil, the DT_50_ values of triasulfuron increased 2.8, 2.7, and 2.1 times compared to non-sterilized unamended soil treated with the herbicide at 2, 10, and 50 mg kg^-1^, respectively (data not shown). These results indicate that microbial degradation played an important role in herbicide degradation, although chemical degradation by hydrolysis was also an important pathway of triasulfuron degradation as stated by other authors (Sarmah et al., 1998; Said-Pullicino et al., 2004).

### Soil Microbial Biomass

**FIGURE 2:**
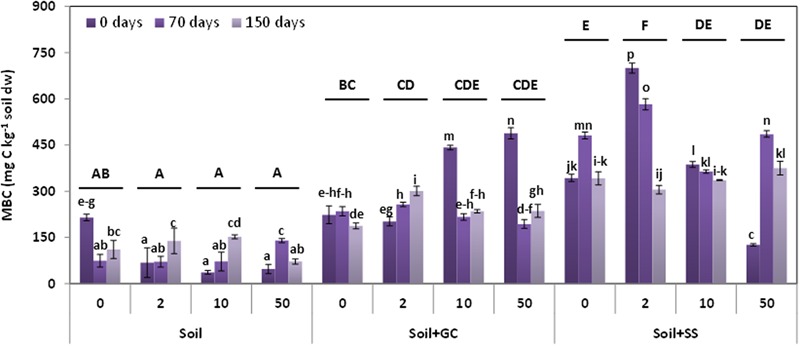
**Soil microbial biomass carbon in unamended and amended soils, untreated (control) and treated with triasulfuron at 2, 10, and 50 mg kg^-1^, at different incubation times.** Bars represent the SD of the mean (*n* = 2). Different lower case letter above the bars denote significant differences (*P* < 0.05) between sampling times for herbicide doses and different upper case letter above the bars denote significant differences (*P* < 0.05) between herbicide doses (mean values) for each soil considering unamended and amended soils jointly.

**Figure 2** shows the results on the microbial biomass (expressed as mg C kg^-1^ soil dw) of unamended and amended soils without triasulfuron (control), and treated with triasulfuron at three doses (2, 10, and 50 mg kg^-1^), and at three different incubation times (0, 70, and 150 days). One-way ANOVA considering all the soils and treatments jointly showed significant differences between the biomass mean values of soils (S < S+GC < S+SS, *P* < 0.001). There were no significant differences between the biomass mean values for the incubation times, or for the doses of herbicide.

However, a two-way ANOVA analysis indicated that interactions between herbicide dose or incubation time and soil samples were significant (*P* < 0.05). The biomass mean values in the unamended soil decreased after herbicide treatment, and they increased after application of triasulfuron at 10 and 50 mg kg^-1^ in S+GC or after triasulfuron application at 2 mg kg^-1^ in S+SS (*P* < 0.05) (soils with the same upper case letter in **Figure 2** were not significantly different).

On the other hand, the biomass mean values were not significantly different over the incubation period in the S soil, but decreased over time in amended soils. Increased biomass values were obtained initially (at 0 days) in S+SS and S+GC soils (*P* < 0.05), although maximum values were obtained at 70 days in S+SS (*P* < 0.1) (treatments with the same lower case letter in **Figure 2** were not significantly different). At time 0 days, biomass decreased in S and S+SS when triasulfuron was applied at the highest dose.

The highest value of microbial biomass for the S+SS soil might be due to the higher OC content (27%) of SS than of GC (8.06%). It has been noted that amendments have a stimulating effect on soil microbial biomass, acting as additional carbon and energy sources (Grenni et al., 2009; Pose-Juan et al., 2015a) and providing additional microorganisms that could affect community dynamics. Furthermore, SS with a lower C:N ratio might be rich in nutrients due to its rapid decomposition, giving rise to higher microbial biomass values (Singh et al., 2016). On the other hand, the S+SS treated with the different doses of herbicide recorded higher biomass values, with the highest value obtained in soil treated at 2 mg kg^-1^, although this peak value was not maintained with incubation time.

It has previously been reported that the use of triasulfuron in unamended soils at the recommended application rate does not affect soil microbial biomass (Lupwayi et al., 2004). However, triasulfuron applied at 10-fold the field dose had a detrimental effect on soil microbial biomass (Sofo et al., 2012), as it does here. The application of other sulfonylurea herbicides or biocides (bensulfuron-methyl, rimsulfuron, or pentachlorophenol) at higher rates than the field rate significantly decreased the soil microbial biomass carbon (Perucci et al., 1999; El-Ghamry et al., 2002; Scelza et al., 2008).

### Soil Dehydrogenase Activity

**FIGURE 3:**
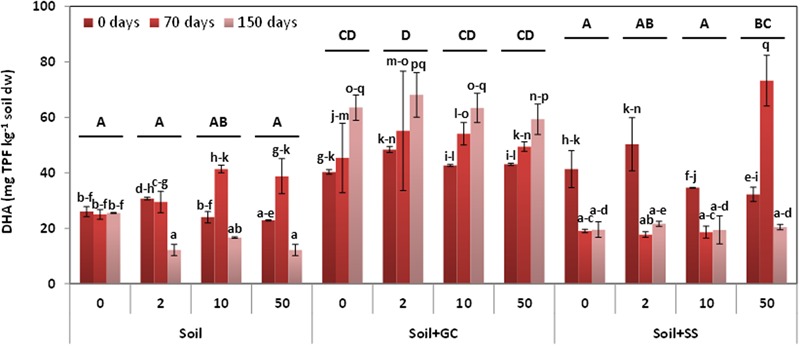
**Soil dehydrogenase activity in unamended and amended soils, untreated (control) and treated with triasulfuron at 2, 10, and 50 mg kg^-1^, at different incubation times.** Bars represent the SD of the mean (*n* = 2). Different lower case letter above the bars denote significant differences (*P* < 0.05) between sampling times for herbicide doses and different upper case letter above the bars denote significant differences (*P* < 0.05) between herbicide doses (mean values) for each soil considering unamended and amended soils jointly.

**Figure 3** shows the results for DHA in unamended and SS- or C-amended soils, untreated (control) or treated with triasulfuron at doses of 0, 2, 10, and 50 mg kg^-1^, and at three different incubation times (0, 70, and 150 days). When considering all the soils and treatments jointly, the results reveal significant differences between the DHA mean values of soils (S < S+SS < S+GC, *P* < 0.001) and incubation times of soils treated with the herbicide (150 days < 0 days < 70 days, *P* < 0.1), but there were no significant differences between the DHA mean values at the different doses applied. However, significant interactions were found between herbicide dose or incubation time and soil samples (*P* < 0.05 and *P* < 0.001), and between herbicide dose and incubation time (*P* < 0.001).

The lowest DHA mean values were obtained in the unamended soil, and they increased in amended soils. The higher DHA mean values were obtained in S+SS soil treated with triasulfuron at 50 mg kg^-1^ and in the S+GC soil treated with the herbicide at 2 mg kg^-1^ (*P* < 0.001) (soils with the same upper case letter in **Figure 3** were not significantly different). On the other hand, the DHA values decreased in S and S+SS soils over the incubation period, although peak values were observed in both soils at 70 days of incubation. In the S+GC soil, the DHA values increased during the incubation for all the herbicide treatments (*P* < 0.001). (**Figure 3**, treatments with the same lower case letter were not significantly different).

The increase in amended soil DHA has been attributed to the higher OC content of these soils and the introduction of new microorganisms with the amendment (Delgado-Moreno and Peña, 2007), and it has been observed in previous works (Moorman et al., 2001; Marín-Benito et al., 2012, Álvarez-Martín et al., 2016).

The herbicide treatment at higher doses than the agronomic dose increased DHA values in S and S+SS. However, the application of herbicides, such as rimsulfuron or pentachlorophenol, inhibited DHA when applied at higher doses than the field rate (Perucci et al., 1999; Scelza et al., 2008). Initially, DHA was inhibited in S and S+SS soils treated with triasulfuron at 10 and 50 mg kg^-1^, indicating that triasulfuron might have a toxic effect on microorganisms, although it was stimulated in these soils at 70 days. At 70 days, triasulfuron concentration in soil decreased to a level that no longer inhibited microbial activity and could be consumed by some of the microorganisms increasing their activity. Pampulha and Oliveira (2006) have found that the combination of bromoxynil and a sulfonylurea (prosulfuron) applied at three increasing doses inhibited DHA, with no recovery over time.

### Soil Respiration

**FIGURE 4:**
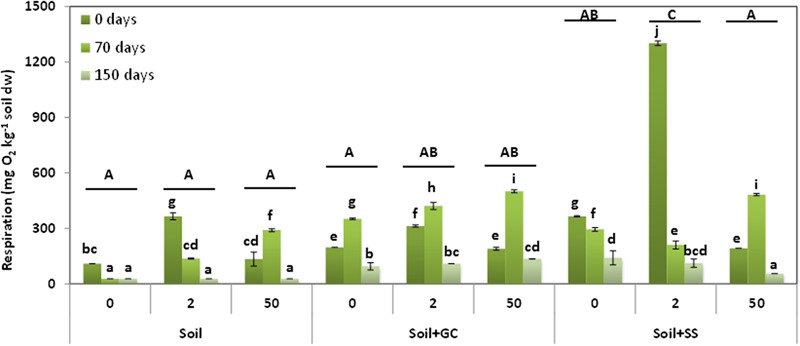
**Soil respiration in unamended and amended soils, untreated (control) and treated with triasulfuron at 2, 10, and 50 mg kg^-1^, at different incubation times.** Bars represent the SD of the mean (*n* = 2). Different lower case letter above the bars denote significant differences (*P* < 0.05) between sampling times for herbicide doses and different upper case letter above the bars denote significant differences (*P* < 0.05) between herbicide doses (mean values) for each soil considering unamended and amended soils jointly.

**Figure 4** presents the soil respiration results in unamended and SS- or C-amended soils, untreated (control) or treated with triasulfuron at doses of 2 and 50 mg kg^-1^, and at three different incubation times (0, 70, and 150 days). When considering all the soils and treatments jointly, the ANOVA revealed significant differences between the respiration mean values of all the soils (S < S+GC < S+SS, *P* < 0.001) and the incubation times of soil treated with the herbicide (150 days < 70 days = 0 days, *P* < 0.001) or doses applied (0 mg kg^-1^ = 50 mg kg^-1^ < 2 mg kg^-1^, *P* < 0.05). Significant interactions were found between herbicide dose or incubation time and soil samples (*P* < 0.05 and *P* < 0.001), and between herbicide dose and incubation time (*P* < 0.001).

The lowest respiration mean values were obtained in S soil and they increased in the amended soils. In S+GC soil, the respiration mean values increased after treatment with the herbicide and in S+SS soil an increased respiration was observed in soils treated with the herbicide at 2 mg kg^-1^ and decreased in the soils treated at the highest dose (soils with the same upper case letter in **Figure 4** were not significantly different). Moreover the respiration mean values decreased in the three soils over the incubation period. The lower values were observed after 150 days of incubation in the soils treated with the herbicide, although a maximum value was observed in the amended soils after 70 days of incubation. Peaks values of DHA were also observed for S and S+SS soils (**Figure 4**, treatments with the same lower case letter were not significantly different).

An increase in soil respiration after the application of amendments to the soil has been previously reported (Perucci et al., 2000; Mattana et al., 2014; Rodríguez-Liébana et al., 2014). Triasulfuron treatment has a significant effect on soil respiration, especially in S+SS treated with a dose of 2 mg kg^-1^. In another study, the application of the sulfonylurea chlorimuron-methyl at three different rates increased respiration during the initial period of incubation (Xu et al., 2014). Scelza et al. (2008) have also reported that the application of pentachlorophenol to unamended or amended soils increases soil respiration.

Respiration values decreased significantly over time. At time 0 days, respiration increased in the soils treated with 2 mg kg^-1^ of triasulfuron, while it decreased in the amended soils treated with 50 mg kg^-1^. At the beginning of incubation, respiration values increased in SS- and GC-amended soils compared to the unamended one. After 70 days of incubation, respiration decreased in the unamended and SS-amended soils treated with 2 mg kg^-1^ of triasulfuron, while it increased in all the soils treated with the highest dose of herbicide. These results could be due to the dissipation of the herbicide, which by this time was over 50%. The herbicide was therefore used less by the microorganisms as a source of nutrients and energy, and by this time the herbicide applied at the highest dose did not have a toxic effect on the soil microbial community.

### Phospholipid Fatty Acid Profile Analysis

**FIGURE 5:**
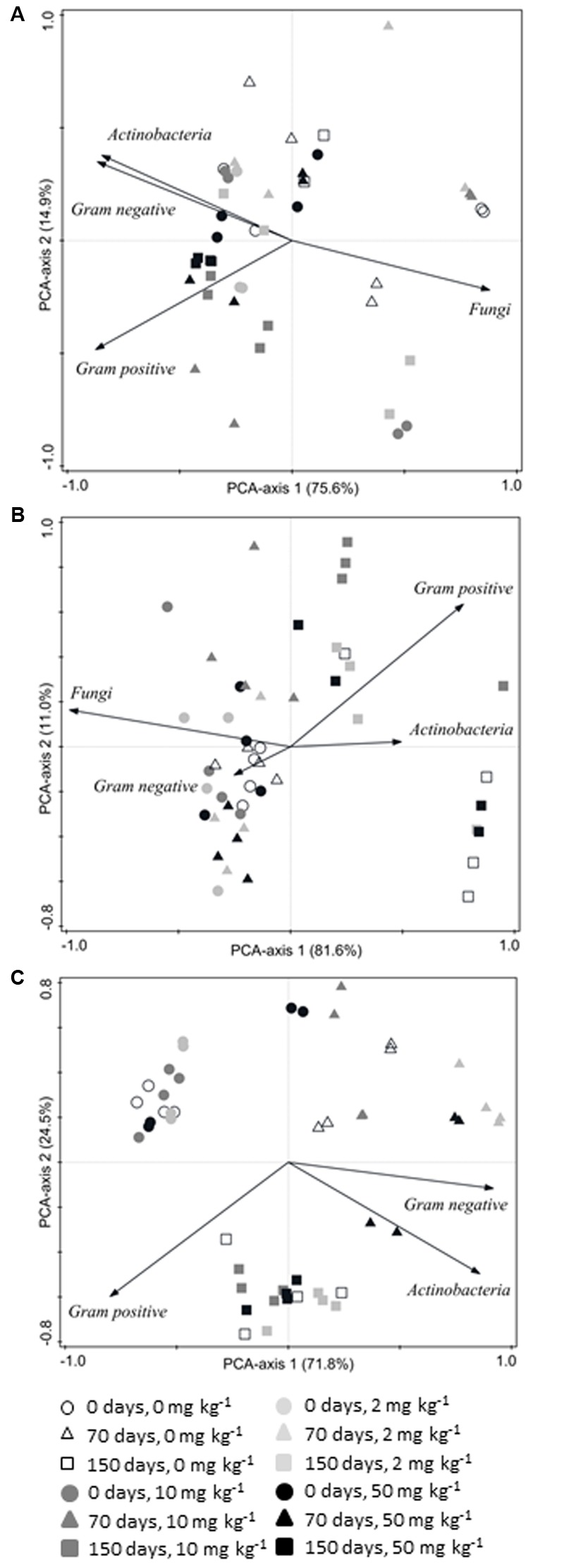
**Biplots representations of the results of PCAs performed on the matrices with relative abundances (%mol) of PLFAs specific for different microbial groups present in (A)** unamended, **(B)** green compost-amended and **(C)** sewage sludge-amended soils. Values on the axes indicate percentages of total variation explained by each axis.

Phospholipid fatty acids profiles are often used to study microbial diversity in complex communities (Zelles, 1999). An indirect analysis (PCA) of the relative abundances (%mol) of PLFAs, specific for Gram-positive and Gram-negative bacteria, *Actinobacteria* and fungi, was used to summarize the variation in the microbial community structure across all 48 soil samples taken from each soil amendment treatment [three sampling times (0, 70, and 150 days) × four triasulfuron doses (0, 2, 10, and 50 mg kg^-1^) × four replicates]. In the case of S, the first two PCA axes explained 90.5% (**Figure 5A**) of the variance in the dataset, while the percentages were 92.6 and 96.3% in the S+GC (**Figure 5B**) and S+SS (**Figure 5C**) soils, respectively. Further examination of the PCA scatter plots, focusing on the arrangement of each sample’s centroids projected *post hoc* in the ordination space, revealed a clear grouping by incubation time in both amended soils (S+GC and S+SS) (**Figures 5B,C**); This result is in agreement with those obtained in studies evaluating effects of other pesticides on microbial communities, in which the elapsed time after the application was also found as decisively influential in shaping the microbial community structure (Karpouzas et al., 2014; Xu et al., 2014; Pose-Juan et al., 2015a). However, such a pattern was not detected in S (**Figure 5A**). Since a relatively low number of PLFAs were detected in the soil used in this study, from a microbiological point of view, it can be classified as poorer than the S+GC and S+SS substrates (16 PLFAs detected in S, against 41 and 35 detected in S+GC and S+SS, respectively; data not shown). Moreover, ranges of total amount of PLFAs (in nmol g^-1^ soil dry weight) throughout all sampling times and triasulfuron doses were 0.3–6.5, 12.6–46.6, and 4.0–58.6 for, S, S+GC, and S+SS, respectively. Thus, it might be hypothesized that the reduced bacterial diversity and biomass in this soil impair the detection of even sharp shifts in the microbial community structure.

In the S+GC (**Figure 5B**), samples collected after 150 days of incubation (squares) are placed on the right side of PCA axis 1, and they are mainly characterized by having higher relative abundances of Gram-positive bacteria and *Actinobacteria* and lower relative abundance of fungi than samples taken at 0 and 70 days (circles and triangles). Gram-positive bacteria, including *Actinobacteria*, are generically known to be good degraders of many complex substrates of carbon (Halverson et al., 2000; de Menezes et al., 2008). This ability might facilitate a steady increase in their relative abundance in a medium rich in cellulose or lignin like compost.

In the S+SS (**Figure 5C**), samples from the three incubation times are clearly separated along the first two PCA-axes. Samples collected at 0 days (circles) were characterized by having the lowest relative abundances of Gram-negative bacteria and *Actinobacteria*, whereas those collected after 70 days of incubation (triangles) had the lowest relative abundance of Gram-positive bacteria. This was probably due to a stimulatory effect by the surplus of organic substrates and nutrients released with the application of SS, since Gram-negative bacteria are fast-growing microorganisms that utilize a range of carbon sources and can adapt quickly to a variety of environmental conditions (Ponder and Tadros, 2002; Bastida et al., 2015).

Redundancy analysis testing for the significance of effects of incubation time, triasulfuron doses, and their interaction on relative abundances (%mol) of PLFAs revealed the statistical significance (*P* < 0.05) of all three factors in the S+SS, and of incubation time and triasulfuron doses in the S+GC; however, triasulfuron doses were the only statistically significant effect (*P* = 0.069) in S (**Table 3**).

**Table 3:** Results of constrained multivariate analyses (RDA) of the effect of sampling time (T), triasulfuron doses (D), and their interaction (TxD) on the relative abundance (%mol)^a^ of PLFAs specific for Gram-positive and Gram-negative bacteria, *Actinobacteria* and fungi in unamended and amended soils.

Explanatory variables	Covariables	Pseudo-*F*	*P-*value^b^	Explained variance (%)^c^
**Soil**				
Sampling time (T)	D	1.0	0.384	0.2
Triasulfuron doses (D)	T	2.2	0.069	7.7
T × D	T, D	1.1	0.354	1.9
**Soil+Green Compost**				
Sampling time (T)	D	42.9	0.001	65.5
Triasulfuron doses (D)	T	3.1	0.007	12.2
T × D	T, D	0.9	0.547	0.0
**Soil+Sewage Sludge**				
Sampling time (T)	D	88.3	0.001	79.9
Triasulfuron doses (D)	T	3.9	0.002	16.4
T × D	T, D	3.1	0.006	22.9

*^a^%mol data were arcsine-transformed prior to RDA analysis. ^b^*P*-values are based on unrestricted Monte Carlo permutation tests (999 permutations). ^c^Explained variance is the sum of all canonical eigenvalues.*

**FIGURE 6:**
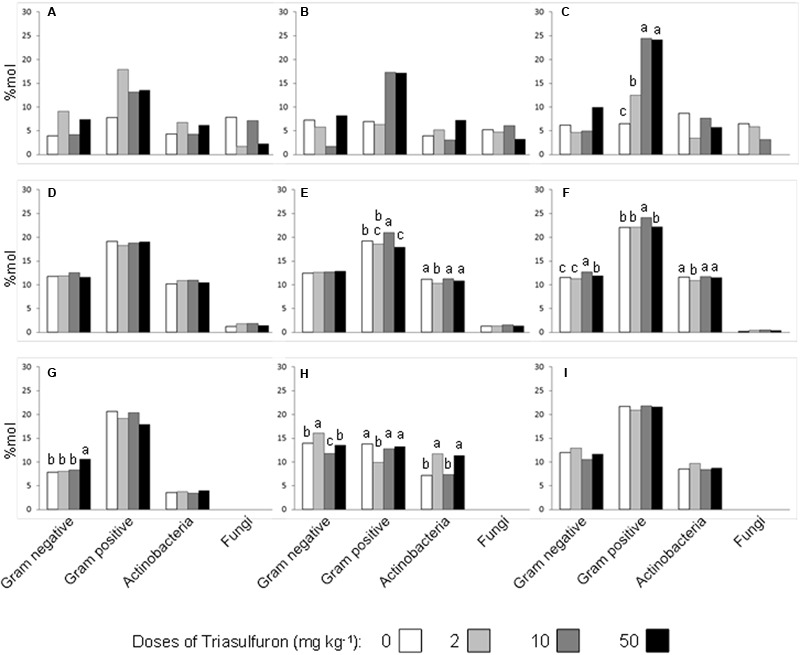
**Relative abundance (%mol) of PLFAs specifically diagnostic of Gram-negative and Gram-positive bacteria, *Actinobacteria* and fungi in unamended soils (A–C)**, green compost-amended soils **(D–F)** and sewage sludge-amended soils **(G–I)** at 0 **(A,D,G)**, 70 **(B,E,H)**, and 150 **(C,F,I)** days after application of four different doses of triasulfuron. Bars with different letters within each bacterial group are statistically different based on LSD test (*P* ≤ 0.05).

In the case of S, a one-way ANOVA on the relative abundances (%mol) of a particular groups of microorganisms showed statistically significant differences only after 150 days of incubation, with the relative abundance of Gram-positive bacteria being significantly increased by the higher doses of triasulfuron (**Figure 6C**). In S+GC soil, triasulfuron did not affect the relative abundance of any bacterial group at the first sampling time (0 days), nor that of fungi at any incubation time (**Figures 6D–F**). After 70 days of incubation, the dose of 10 mg kg^-1^ was found to have a positive significant effect on the relative abundance of Gram-positive bacteria, with a significant lowering of the relative abundance of *Actinobacteria* by the 2 mg kg^-1^ dose of triasulfuron (**Figure 6E**). At 150 days, the 10 mg kg^-1^ dose of triasulfuron tended to increase the relative abundance of both Gram-positive and Gram-negative bacteria, as well as that of *Actinobacteria* (**Figure 6F**). Finally, in contrast to the S and S+GC soils, triasulfuron doses had no significant effect on the relative abundances of the microbial groups at 150 days in the S+SS soil (**Figure 6I**). Furthermore, in contrast to the S and S+GC soils, fungal-specific PLFAs were not detected in the S+SS soil (**Figures 6G–I**). There is no obvious explanation for this fact, but it might reflect the presence of some fungal inhibitory substance in the SS used, as it has been reported in the case of bacteria (Malara and Oleszczuk, 2013). At the first sampling time, the application of the highest dose of triasulfuron (50 mg kg^-1^) led to a significant shift in the relative abundance of Gram-negative bacteria (**Figure 6G**). Such a shift was also observed at 70 days, albeit with the application of 2 mg kg^-1^ of triasulfuron instead of 50 mg kg^-1^ (**Figure 6H**). However, the 2 mg kg^-1^ dose of triasulfuron significantly reduced the relative abundance of Gram-positive bacteria at 70 days, while the relative abundance of *Actinobacteria* at 70 days appeared to be erratically influenced by the doses of triasulfuron (**Figure 6H**).

Therefore, the effects of triasulfuron doses and incubation times on relative abundance of the four microbial groups were no consistent for any microbial group throughout the three soil treatments, although some statistically significant differences were observed (**Figure 6**). Lupwayi et al. (2004) reported that triasulfuron and other herbicides applied once at recommended rates did not have significant or consistent effects on microbial biomass (microbial C) or diversity. Other authors found slight effects of triasulfuron and other three sulphonylureas at normal field doses on soil microbial biomass and its biochemical activities, although all the four herbicides exerted a stronger detrimental effect at 10-fold the field dose (Sofo et al., 2012). We can, however, conclude that triasulfuron, under the conditions and at the doses tested in our study, did not exert notable modifications on microbial community structure at the level we could detect.

## Author Contributions

Designed experiments: MR-C and MS-M. Performed experiments: EP-J, MR-C, and JI. Analyzed results: MR-C, MS-M, EP-J, and JI. Wrote the manuscript: EP-J, MR-C, MS-M, and JI.

## Conflict of Interest Statement

The authors declare that the research was conducted in the absence of any commercial or financial relationships that could be construed as a potential conflict of interest.

The reviewer CP and handling Editor declared their shared affiliation, and the handling Editor states that the process nevertheless met the standards of a fair and objective review.
